# Valuing Australian football league draft picks

**DOI:** 10.1371/journal.pone.0292395

**Published:** 2023-10-03

**Authors:** Jemuel Chandrakumaran, Mark Stewart, Preety Srivastava

**Affiliations:** 1 Institute for Health and Sport (IHES), Victoria University, Melbourne, Australia; 2 School of Economics, Finance and Marketing, RMIT University, Melbourne, Australia; 3 Athletics Australia, Albert Park, Australia; Privatuniversität Schloss Seeburg: Privatuniversitat Schloss Seeburg, AUSTRIA

## Abstract

To ensure uncertainty in match outcomes, professional sporting leagues have used various competitive balance policies, including player salary caps, revenue sharing among teams and player drafts. The Australian Football League (AFL) introduced a player draft in 1986, and to refine its operation, a draft value index (DVI) was introduced in 2015. The DVI allocates a numeric value to each individual player draft pick, with these values determined by the AFL using historic player compensation or wage and salary data. The AFL DVI plays an essential role in the operation of its player draft; however, other research has questioned the validity of such indexes. This paper aims to produce an alternative to the AFL DVI. The former index uses career compensation as the determinant of value, whereas we use other measures of player performance. First, various models were developed to predict on-field performance, such as games played (both in a recruit’s career and season) after a draftee was selected for the first time by a team. This was then retrofitted to the pick used to select these draftees to create the new DVIs. Even though the predicted DVI followed an inverse monotonic function like the existing index, the decline in value for the DVI produced here was less steep, unlike the AFL’s. This allowed us to conclude that players’ salaries did not always strongly correlate to performance. The change in performance between players selected at different points in the draft did not vary as much as their wages. Though this scheme is applied to the AFL, the underlying concept could be directly exported to other player drafts.

## Introduction

Rottenberg [1] applied the theory of competitive balance within the sporting arena and surmised that ‘no team can be successful unless its competitors also survive and prosper’. Should the market for talent (i.e., players) be left unrestricted, wealthier teams will continue to acquire the best players, depleting this common resource. This, in turn, will reduce the uncertainty of match outcomes, as the best players will all be employed by a collection of wealthy teams, and this may ultimately reduce spectator appeal. This proposition has been the subject of much academic research, with examples including Borland et al. [2], Forrest [3] and Fuller et al. [4] whilst some have even looked at the incentives to compromise on competitive balance when the sport in question has an intermingled domestic and international league [5, 6]. Ideally, leagues would like ‘every “well-run” club to have a regularly recurring reasonable hope of reaching postseason play’ [7].

Understanding this concept, professional sporting leagues have introduced many policies such as revenue sharing among teams and salary caps to induce competitive balance (i.e., to preserve the common resource). Concurrently, various measures have also been developed to evaluate the validity of these policies [8]. However, the effects of these policies may not always produce the desired outcome of an even competition. For example, studies have shown that salary caps may be ineffective [9–11]. Player drafts are also commonly used for the same purpose and deny overbidding for amateur talent. Whilst the draft may sometimes fail to achieve its goals in major competitions [12–15], practitioners have suggested that an accurate value function for draft picks that corrects trades, may improve overall competitiveness [16]. This paper aims to evaluate the value of such picks within the Australia Football League (AFL). Currently there is a league endorsed draft value index (DVI) that was created by retrofitting career compensation of various players selected at different points in the draft [17]. However, as player compensation is not generally determined by marginal productivity [18], the trade correction referred to earlier will fail to materialise. For the draft to be effective it important that players selected through the draft represent their pick value as this will reshape the rosters of each team cyclically [19]. This study will first model player performance using various alternative measures considering factors such as race, physical metrics and drafting scenarios. The resulting models will then be used to predict player performance, which will be fitted against draft pick numbers to construct an alternative DVI. This alternative DVI is compared with the one used by the AFL.

## AFL draft

With respect to many indicators, such as television ratings, viewership and revenue generation, the AFL is the most popular professional team sporting league in Australia [20]. However, in the late 1970s and 1980s, many clubs fell into dire financial situations, partly due to exhaustive player bidding wars and consequent dominance exerted by a few wealthy clubs [21]. As a result, the league introduced the national player draft in 1986 as one of many competitive balance policies [13]. The selection order within the draft was based on a conventional reverse order season standing system like those observed in major North American drafts. Inherently, this means that a team that finishes last in the season immediately before the draft will have the first selection, followed by the second-last placed team until a complete round of selections is complete. This process recurs continuously for approximately four more rounds in the current version of the draft. Furthermore, the AFL allows teams to draft players who have previously played in the league as well (provided they are delisted by their former clubs and have no restrictions).

The league has continued to refine these competitive balance policies, and in 2015, introduced the DVI (the acronym DVI is used in the AFL, while most North American leagues including the National Football League (NFL) and National Hockey League (NHL) refer to it as the Pick Value Chart (PVC)), which will be the focus of this paper. Through this mechanism, the league administers a numerical value to each individual draft pick using historical player salary data. This is used for a number of purposes, including providing priority access for clubs to players through the father–son rule, and players from within club-academies, as well as imposing draft penalties on clubs for misconduct.

### Father-son (F/S) rule

The father–son rule has been in effect within the league since 1949. The rule means that teams have the choice to recruit the sons of former players, which portrays and markets the AFL as a family-oriented legacy affair [22] for a nostalgic effect. It is one of two priority access selections (PAS). The rules pertaining to the eligibility of players have changed over time, where the current system requires the father to have played a minimum of 100 games for the club considering recruiting the son.

Before 1997, players eligible under this criterion would be taken up by their prospective teams before the beginning of the draft itself. However, in 1997, the AFL decided to incorporate this rule within the draft, causing teams to effectively pay a price (by using a pick) for their priority access to such players. These nominations would usually happen right after post-season play but before the trading period and the draft, giving teams little to no time to make informed decisions. Under these stipulations, teams would generally use their next available selection (this would be the pick that the team has in its possession that it can use next; for example team A might have picks 1 and 19, but if they have used pick 1, their next available pick will be 19) to draft eligible players (prior to 2007, teams nominating F/S players would only give up their third-round pick and not their next available pick). This process did not accurately display the market value of these players [23].

### Club-academy (C/A) rule

The club academy principle was first introduced in 2010 after revising the existing scholarship system, where the four northern clubs (Sydney, Greater Western Sydney (GWS), Brisbane and Gold Coast) were allocated a geographical zone to set up and train junior footballers in these areas where the most popular football code is rugby league rather than Australian football. The intention was to promote the sport by guaranteeing amateurs a pathway into the sport with their hometown. The clubs that ran these academies were further granted priority access (not exclusive) to amateurs who graduate from them through the national draft. The first academy graduates were eligible for the draft in 2011, and parent clubs could acquire these amateurs with their next available pick by nominating them before the beginning of the draft, akin to F/S players. This caused a similar dilemma to that observed in the F/S scenario, where amateur players were selected at picks that were not consistent with their draft value.

### Draft Value Index (DVI)

The main problem faced in both F/S and C/A situations was the indivisibility of potential draftees or draft picks. This meant that the priority access club had the opportunity of procuring F/S or C/A players by using their next available pick rather than matching the pick of the competing club. Even if the priority access club wanted to compensate the competing club for losing the player in question, there was no standard measure to equate the lost value. Moreover, the draftee’s market value was withered down to his subsequent selection compared to the initial bid by the competing club (assuming that there is an indirect relationship between draft picks and value).

To circumvent this problem, the league introduced the DVI in 2015. Official AFL communications specified that the DVI was obtained through fitting salaries paid to draftees at various points over 15 drafts (2000 to 2014), ‘which assigns a relative points value for each pick in the National Draft’ [17]. The choice of player salaries was justified because it was ‘an indicator of relative market value of players at each draft pick’ [17, 24]. Upon further investigation, it is evident that the DVI follows a conventional log normal wage function [19]. Akin to the PVC in the NFL, the DVI declines exponentially in the first-round selections, while teams often use it as a guide in trading picks during the draft. However, contrary to the PVC and its counterparts in the United States, the DVI is the only league-administered draft index that is used to allocate resources pertaining to the policies tied into the draft. In doing so, the league has been able to address the continuing problem of value. The DVI has inherently served both as the currency to use in bidding transactions and as the price of a pick, equating itself to money when clubs trade players and draft picks.

### Bidding system

As mentioned earlier, the primary reason for the existence of the DVI is because of the new bidding system introduced in 2015 for F/S and C/A players. Before 2015, for both of these recruitment concessions, teams were allowed to nominate players prior to the draft and select them with their next available draft pick, should another rival team bid for them earlier on in the draft.

Under the new rules, any bid by a rival club for either an F/S or C/A player has to be met in kind by the priority access club with a discount. As a rule, teams matching the bids for F/S and C/A players are entitled to a 20% discount on the DVI points of the competing bid they are required to match up until pick 18. Any selections made after this is given a constant discount of 197 points, which is equivalent to 20% of the eighteenth pick’s DVI points. The 20% discount on the DVI awarded to clubs that are making use of either the F/S or C/A rules is an arbitrary amount. This discount is because the AFL still wants clubs to use these rules, but they do not want them to gain too much of an advantage from them.

Moreover, the new bidding system is conducted during the draft, unlike the earlier method, which was held straight after the post-season games, giving the nominating and bidding teams the ability to analyse their draft positions and needs effectively. The following example will put this into perspective.

In 2014, before the introduction of the DVI, Jack Steele was a C/A player from the GWS club and was eligible to be drafted. North Melbourne had the fifteenth pick in the draft and nominated Steele as their choice. However, GWS invoked the C/A rule to select Steele. Under the rules in 2014, GWS could match this bid with their next available pick, which at the time was the twenty-third selection. Thus, North Melbourne was forced to select another player at pick 15, while GWS acquired Steele with pick 23. This happened before the draft, after post-season games had been completed, but before the trade period. Due to this, GWS could not effectively analyse if they needed Steele, as there was a possibility that their needs would differ following the trade period. Under the new bidding system used since 2015, GWS would still be eligible to invoke the C/A rule privileges during the draft and not before but would have to provide more than just the twenty-third pick to do so (refer to Table 1 for an illustration of the pick movements).

**Table 1:** Comparison between old and new bidding systems.

Old System			New System		
Pick	Team	Player	Pick	Team	Player
1–14			1–14		
15	North Melbourne		15	Greater Western Sydney	Jack Steele
16	Essendon		16	North Melbourne	
17	Sydney		17	Essendon	
18	Carlton		18	Sydney	
19	Essendon		19	Carlton	
20	St. Kilda		20	Essendon	
21	St. Kilda		21	St. Kilda	
22	Melbourne		22	St. Kilda	
23	Greater Western Sydney	Jack Steele	23	Melbourne	
24	Greater Western Sydney		24	North Melbourne	
25	North Melbourne		25	Western Bulldogs	
26	Western Bulldogs		26	Western Bulldogs	
27	Western Bulldogs		27	Greater Western Sydney	

Since North Melbourne bid for Steele with pick 15, GWS would have to match the DVI points of the fifteenth pick with their available draft picks, although with a 20% discount because Steele was from their C/A. The fifteenth pick is worth 1,112 points per the DVI, but the 20% discount reduces this to 890 points. At this stage in the draft, GWS’s next available picks were 23 and 24. The twenty-third pick is valued at 815. In losing its position at 23, GWS would move to 15, while effectively moving all other selections from picks 15 to 22 one spot down. That is, North Melbourne, who had the fifteenth selection, would now be allowed to re-select another player at pick 16, with the rest of the drafting order to follow suit.

However, GWS would not fulfill their obligation in matching the bid made by North Melbourne at pick 15. They would still owe 75 points (1,112*20%– 815 = 75). These 75 points would be deducted from their next available pick, which would be the twenty-fourth selection, valued at 785 points. In doing so, GWS would be left with 710 points, which is roughly equivalent to the twenty-seventh selection. Thus, GWS’s twenty-fourth pick would be moved down to 27, while the picks between 24 and 27 would move up one step.

Overall, the aim of the DVI would be achieved here in terms of making sure that teams bidding for F/S and C/A players pay something close to market value for their picks. As the example suggests, Steele would be drafted to an AFL club as a first-round draft pick (aligned with his market value), unlike the second-round selection he was recruited with under the old bidding system.

The new system allows the priority access clubs to use up all their DVI points for the current year in bidding for F/S and C/A players while also allowing them to go into a deficit to a total of 1,723 points. The deficit is recovered from the team’s following year DVI points at the same round in which the deficit was created. For example, if GWS ran up a deficit of 100 points in selecting F/S or C/A players in the current year for a second-round pick, 100 points would be deducted from their next year’s point balance from their second-round pick.

## Prevailing issues

The changes to the bidding system and the introduction of the DVI have shifted the strategy behind preparing for the draft. Previously, clubs traded picks based on acquiring the best talent early on. Now, the bidding system encourages clubs to trade for points and not just picks, as their priority access gives them the opportunity to recruit the player they desire, as long as they have the points to pay for them. Second, there is the potential for false bidding. Although never proven, bidding teams do have the ability to engage in false bidding. Such a scenario would be a colluded effort by multiple teams to nominate multiple F/S or C/A players for one rival team in the first round. In doing so, they will deplete the points balance of the priority team early on, preventing them from running for other potential draftees.

Moreover, the choice of player payments to determine the value of the DVI raises further questions. First, as draftees in the AFL are not strictly amateurs (i.e., players who have played before in the AFL are also allowed to enter the draft, which is not the case in any North American draft), their pay reflects their experience [18] and star power [25–28], independent of the pick used to select them in the draft. Conversely, struggling teams with fewer senior players with room in their salary cap [29] can spend more on fresh recruits. Further, as early selections do tend to gain more tenure and game time irrespective of their talents [30, 31], a case could be made against the use of player payments as the determinant of the DVI.

To circumvent this, Mitchell et al. [32] and Stewart et al. [23] use games played and champion data playing ranking points (Champion Data (CD), in conjunction with the school of mathematics at Swinburne University, introduced the CD player ranking points as a proprietary method of objectively evaluating player performance by assigning values to a range of on field statistics that are important for team success) as alternative measures of performance to value picks within the AFL, consistent with studies in other leagues [16, 33–35]. Their findings characterise further inefficiencies within the AFL caused by the F/S rule and the selection of Aboriginal and Torres Strait Islander players, which is unique to the league itself. Moreover, they suggest that other factors such as player physique, state of origin and amateur league can further skew the performance variable, reducing the impact to playing time by the draft pick number alone. Other models developed using the contribution to the margin of victory [31] and survival analysis [36] reaffirm that player salary and performance are not necessarily perfectly correlated. The objective of the DVI is to improve the workings of the AFL draft, thereby assisting the league to achieve competitive balance. If the DVI does not accurately reflect player performance the draft’s ability to achieve an even competition will be compromised.

The aim of this paper is to estimate alternative models for player performance using games played, time on field and Brownlow Medal Votes (a system where umpires vote for the three best players on the field per game). We then construct respective DVIs by predicting players’ performance. Finally, we compare the AFL DVI to these alternate indices.

## Data

A database of all selections in the AFL national player draft from 2003 through to 2016, supplemented by their performance in the league’s home and away seasons (H/A) from 2004 to 2017 was constructed. Although the national draft has been operational since 1986, this study only considers players selected from 2003 onwards. A multitude of factors has led to the decision to use this cut-off point including accuracy and completeness of the data. In the period leading up to 2003, the number of players selected in each draft varied considerably due to the introduction of new teams and foundation selections. Post-2003, the data obtained (and used in this paper) have been more consistent.

Unlike most professional sporting leagues that use a draft, the AFL also allows players who have played before in the league to be selected in the draft. To avoid biases created by such players, any draftee who had played before in the AFL or was a rookie listed player and elevated as a senior player through the draft has been excluded from this dataset. Furthermore, as the main objective of this paper is to compare the player performance based DVI against the existing AFL DVI which terminates at pick 73, any player selected after pick 73 is also excluded from the dataset. Coincidentally, most observations that are excluded due to the ‘played before’ exception forms a major part of the second exclusion filter. This leaves 907 cross-sections (players) in the career aggregate dataset or a panel of 4,777 player seasons. This sample is used to estimate the models defined of draftees selected from 2003 to 2016, with their respective performance from seasons 2004 to 2017. Table 2 describes all variables used in the study.

**Table 2:** Description of variables.

	Description
**Dependent Variables**	
Games Played ^a^^,^^b^	The number of regular season games played throughout a draftee’s career. Post-season matches are not included as they may create an inherent bias towards players from successful teams who get to play more games.
	A normal AFL regular season consists of 23 rounds, of which any team will play 22 home and away games and have one round of rest, known as a ‘bye’.
Time on Field ^a^^,^^b^	The number of minutes spent on field in the games played above.
Brownlow Votes ^a^^,^^b^	Brownlow votes accumulated throughout the above games.
**Independent Variables**	
Pick ^a^^,^^b^	Selection number in the AFL national player draft used to obtain the player.
Drafting Age ^a^^,^^b^	Age of the draftee as of 31 December in the year when he was drafted.
Season Age ^b^	Age of the draftee as of 31 December in the year in which the seasonal data are recorded.
Indigenous ^a^^,^^b^	Dummy variable = 1 if Indigenous player, 0 otherwise.
Father–son (F/S) ^a^^,^^b^	Players drafted under the F/S rule were distributed under three groups.
	≤2006 = Team that chose F/S players in or prior to the 2006 draft were only meant to compensate the competing bid for the same player with a third-round pick.
	2007 ≤ 2014 = F/S players chosen between 2007 and 2014 are those who were affected by the 2007 rule change whereby teams were meant to match a bid for the same player with a pick in the same round.
	≥2015 = The DVI was introduced in 2015, whereby teams choosing F/S players had to compensate competing bids with equivalent draft points as described in the bidding system sub section.
	A dummy variable was created for each group such that the variable = 1 if player belonged to the respective group, 0 otherwise.
Club academy (C/A) ^a^^,^^b^	Players drafted under the C/A rule were distributed under two groups.
	≤2014 = C/A players chosen in or prior to the 2014 draft were meant to compensate a competing bid for the same player using a pick in the same round.
	≥2015 = The DVI introduced in 2015, enforced the same bidding procedure observed by F/S players.
	A dummy variable was created for each group such that the variable = 1 if player belonged to the respective group, 0 otherwise.
Height ^a^^,^^b^	Height in centimetres.
Weight ^a^^,^^b^	Weight in kilograms.
Position ^a^^,^^b^	The most common position played by the player in his career (Defender, Forward, Midfielder and Ruckman). The categorical variable is converted into 4 respective dummy variables defined as Defender = 1, 0 otherwise (base category), Forward = 1, 0 otherwise, Midfield = 1, 0 otherwise, Ruckman (a key centre player in Australian football who contends for the ball during stoppages) = 1, 0 otherwise.
Drafting Team ^a^^,^^b^	Team that drafted the player. A dummy variable was created for each team such that the variable = 1 if player belonged to the respective team, 0 otherwise.
Amateur League ^a^^,^^b^	The amateur league from which the draftee is recruited.
	A dummy variable was created for each Amateur League such that the variable = 1 if player belonged to the respective league, 0 otherwise. The four leagues referred to here are TAC Cup, SANFL, WAFL, and others.
Traded ^b^	Dummy variable = 1 if the draftee was traded to another team at the beginning of the season, 0 otherwise.
Same Team as Drafting Team ^b^	Dummy variable = 1 if the team that the draftee is listed in the season is the same as the team that drafted him, 0 otherwise.

^a^ Used in the career model

^b^ Used in the seasonal model

Our primary outcomes of interest to measure player performance are 1) games played, 2) time on field and 3) Brownlow Medal Votes (BMV) for all regular season games. These variables are restricted to regular season home and away games to ensure each player is comparable irrespective of the teams they played for (as the number of games played by draftees in teams that qualify for the post-season will be higher).

The choice of the three performance variables is based on relevance and controls of similar work. Previous studies indicate that games played (and time on field) cannot be used to determine performance because it may include potential decisional biases contrary to the nature of the sport. Hence, many authors have chosen games started [35], ranking points [23] and contribution to the margin of victory [31]. Studies that use games played have described it as a determinant rather than an outcome or measure of draftee performance [37]. Chandrakumaran [36] uses this in a survival analysis context and shows that the use of games played results in a slowly faltering DVI, unlike the AFL DVI, which drops at an exponential rate. In this paper, we also use BMV as a measure of performance. BMV does not only reflect the player’s selection by the team but also his performance thereafter as adjudged by an umpire. Descriptive statistics of the performance measures and all continuous explanatory variables used in the study are presented in Table 3. On average, draftees play 53.44 games during their careers. The seasonal indicators show that, on average, draftees play 4.15 seasons with 10.15 games per season.

**Table 3:** Descriptive statistics of continuous variables.

Variable	Mean	Median	Min	Max	Std. Dev.
Career Games	53.44	28	0	260	60.68
Career Time on Field (in minutes)	3,434.71	1,703.20	0	18,151.20	4,056.53
Career Brownlow Votes	6.71	0	0	181	19.46
Season Listed Games	10.15	9	0	22	8.11
Season Listed Time on Field (in minutes)	652.31	560.80	0	1,758.40	551.94
Season Listed BMV	1.28	0	0	36	3.49
Season Age	22.24	22	18	35	2.93
Season Number	4.15	3	1	14	2.84
Draft Age	18.18	18	17	26	1.11
Season Weight	84.95	85	63	116	8.13
Season Height	188.46	188	167	210	6.91
Career Average Weight	84.93	84	63	112	7.86
Career Average Height	188.25	187.88	167.81	208.88	6.84

To account for seasonal effects in a draftee’s development, Table 4 displays the performance of all draftees since they were drafted (a proxy for experience). The first row shows that out of the 2004 to 2017 players, 906 were listed in their first year after being drafted. On average, they are 19.19 years old, selected with pick number 34, and have played 4.93 games (out of a possible 22). Given the decreasing number of players being listed in a team for every additional year, less confidence can be placed on values beyond the tenth season and much less for those under the special categories.

**Table 4:** Summary statistics by experience.

Years since first drafted	All Players					
	No. of listed players	Averages				
		Age	Pick	Games	Time on field	Brownlow votes
1	906	19.19	33.80	4.93	286.64	0.16
2	829	20.16	33.38	7.75	470.43	0.38
3	687	21.14	31.57	9.83	622.64	0.80
4	571	22.11	30.18	11.76	759.08	1.53
5	448	23.06	28.48	12.56	822.82	1.76
6	370	24.03	27.82	13.22	878.51	2.20
7	288	24.99	26.84	13.78	915.68	2.29
8	225	25.94	26.14	14.12	943.10	2.74
9	171	26.92	25.06	14.74	987.41	3.06
10	125	27.92	25.33	14.65	992.65	3.37
11	87	28.84	26.38	13.97	942.25	2.55
12	45	29.58	21.69	13.16	881.48	3.09
13	23	30.57	21.91	12.39	842.19	1.78
14	6	31.33	33.67	10.83	720.27	0.83

**Fig 1:**
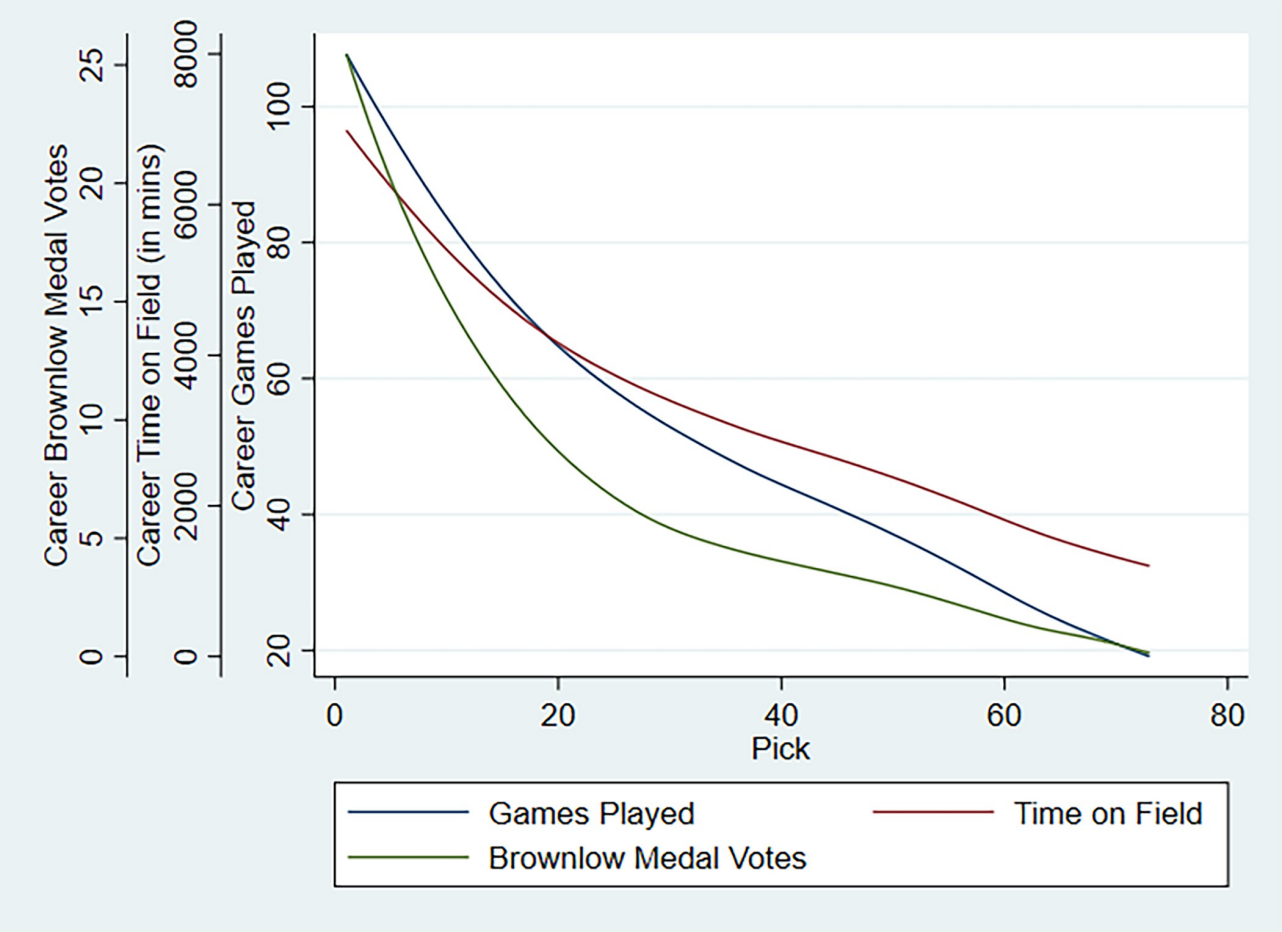
Observed career performance against draft number.

As a precursor to the econometric models, in Fig 1, the three measures of career performance are plotted against pick number using a Locally Weighted Scatterplot Smoothing (LOWESS) model. Relative to the more objective measures, that is, games played and time on field, the trend line of BMV appears to be closer to the AFL DVI. This could suggest that even though game time might equitably distribute among players, their value during that period is represented more by the subjective BMVs they acquire instead.

## Empirical specification and estimation

We specify separate models for career performance and seasonal performance. The choice to use the entire career to create the DVI was based on two key reasons. Firstly, young amateur players who get drafted only play a small number of games in their initial years and peak around season eight or nine (as shown in Table 4). Secondly, a majority of players in the AFL remain with the team that drafted them as they usually derive high levels of performance with them [31]. Together a case could be made that a team’s investment in draft picks would reap meaningful outcomes (i.e., winning seasons) in the long term.

Each of these models is estimated for three different measures of player performance: a) number of games played, b) time on field and c) BMVs. The equation used here was partly built in accordance with the empirical model specified in Stewart et al. [23]. Since the purpose of the model is to predict players’ performance and depict the relationship between pick and predicted performance, we do not include pick as a predictor of performance. In particular, the model is specified as:

$${Career\;Performance}_{i}=\beta_{0}+\beta_{1}Drafting\;{Age}_{i}+\beta_{2}{Indigenous}_{i}+\beta_{3}{FatherSon(\leq 2006)}_{i}+\beta_{4}{FatherSon(2007-\leq 2014)}_{i}+\beta_{5}{FatherSon(\geq 2015)}_{i}+\beta_{6}{ClubAcademy(\leq 2014)}_{i}+\beta_{7}{ClubAcademy(\geq 2015)}_{i}+\beta_{8}{Height}_{i}+\beta_{9}{Weight}_{i}+\beta_{10}{Position(Forward)}_{i}+\beta_{11}{Position(Midfielder)}_{i}+\beta_{12}{Position(Ruckman)}_{i}+{\beta_{13}Drafting\;Team}_{1i}\ldots +{\beta_{29}Drafting\;Team}_{17i}+\beta_{30}{Amateur\;League}_{1i}\ldots +\beta_{32}{Amateur\;League}_{3i}+\varepsilon_{i}$$ (1)

where *Career Performance_i_* is the career-long aggregated performance of the *i*^*th*^ player. In terms of explanatory variables, the type of selection (with different categories for varying rules of father-son and club academy selection), player weight, height, year when drafted, race, position played, amateur league and recruiting team are used.

Unfortunately, with career aggregates, it is not possible to include in-season anomalies such as injuries, not being listed to play and varying career lengths (48% of the sample is still actively playing the league as at 2017). Thus, another model is specified for the seasonal performance of draftees after they were inducted into the AFL, similar to the model defined by Mitchell et al. [32] and Stewart et al. [23]:

$${Seasonal\;Performance}_{it}=\beta_{0}+\beta_{1}Drafting{\;Age}_{i}+{\beta_{2}{Season\;Age}_{it}+\beta_{3}{{Season\;Age}^{2}}_{it}+\beta }_{4}{Indigenous}_{i}+\beta_{5}{FatherSon(\leq 2006)}_{i}+\beta_{6}{FatherSon(2007\leq 2014)}_{i}+\beta_{7}{FatherSon(\geq 2015)}_{i}+\beta_{8}{ClubAcademy(\leq 2014)}_{i}+\beta_{9}{ClubAcademy(\geq 2015)}_{i}+\beta_{10}{Height}_{it}+\beta_{11}{Weight}_{it}+\beta_{12}{Same\;Team\;as\;Drafting\;Team}_{it}+\beta_{13}{Traded}_{it}+\beta_{14}{Position(Forward)}_{it}+\beta_{15}{Position(Midfielder)}_{it}+\beta_{16}{Position(Ruckman)}_{it}+{\beta_{17}Drafting\;Team}_{1i}\ldots +{\beta_{33}Drafting\;Team}_{17i}+\beta_{34}{Amateur\;League}_{1i}\ldots +\beta_{36}{Amateur\;League}_{3i}+\varepsilon_{it}$$ (2)

where *Seasonal Performance_it_* is the performance of the *i*^th^ player at time *t*. In addition to the explanatory variables in Eq 1, four new variables are added to the model. Specifically, a quadratic specification of season age is included to account for the non-linearity in age-performance relationship. The model also controls for indicators of whether a draftee is traded at the beginning of the season in question (= 1, 0 otherwise) and whether the player is listed in the same team as the team that initially drafted him (= 1, 0 otherwise). For example, previous studies have found that players play more frequently in the team that initially recruited them [31].

### Career performance model

As a starting point, an Ordinary Least Squares (OLS) model for all three performance indicators (games played, time on field and BMVs) is estimated using Eq 1 (Table 5). The results show that none of the amateur league variables is statistically significant, implying that a player’s amateur career background does not determine his performance in the AFL. The same can be said about the player position variables, except for midfielders who obtain more games (13.57), game time (712.60 minutes) and BMV (11.93), relative to defenders (the reference category).

**Table 5:** Career performance model regression results.

Variables	Ordinary Least Squares					
	Games Played (1)		Time on Field (2)		Brownlow Votes (3)	
	Coefficient	Std. Err.	Coefficient	Std. Err.	Coefficient	Std. Err.
Drafting Age	–3.22*	1.88	–212.60*	125.60	–1.51**	0.61
Indigenous	33.59***	9.01	2,268.00***	603.00	4.90*	2.90
Father–son						
≤ 2006	21.63	15.21	1,455.00	1,018.00	4.94	4.90
2007 ≤ 2014	–4.72	14.77	–329.00	988.60	0.68	4.76
≥ 2015	–39.59	29.25	–2,517.00	1,958.00	–8.14	9.43
Club academy						
≤ 2014	–25.03	23.08	–1,548.00	1,545.00	–4.51	7.44
≥ 2015	–28.83*	17.28	–1,855.00	1,157.00	–3.23	5.57
Height	–3.06***	0.51	–188.80***	33.78	–0.58***	0.16
Weight	3.80***	0.42	255.30***	28.19	0.84***	0.14
Position Played ^a^						
Forward	–5.55	4.93	–472.80	330.10	2.00	1.59
Midfielder	13.57***	5.13	712.60**	343.50	11.93***	1.65
Ruckman	–8.72	9.74	–1,115*	651.70	–2.47	3.14
Drafting Team ^b^						
Brisbane	0.40	11.05	65.96	739.80	–5.22	3.56
Carlton	–8.59	11.47	–530.40	767.80	–0.68	3.70
Collingwood	–11.20	11.62	–571.70	778.00	–3.22	3.75
Essendon	2.58	11.27	270.40	754.20	–2.91	3.63
Fremantle	–10.55	11.46	–658.80	766.90	–2.21	3.69
Geelong	–14.80	11.41	–951.20	764.00	–3.70	3.68
Gold Coast	–10.54	13.60	–567.00	910.40	–4.63	4.38
Greater Western Sydney	–8.92	13.28	–515.60	888.70	–4.62	4.28
Hawthorn	6.45	11.79	569.40	789.40	0.88	3.80
Melbourne	2.67	11.31	311.00	756.80	–4.12	3.64
North Melbourne	–6.43	11.45	–403.80	766.80	–2.67	3.69
Port Adelaide	–2.08	11.40	–54.14	762.90	–3.15	3.67
Richmond	1.47	11.39	207.30	762.20	0.94	3.67
St. Kilda	0.44	12.19	124.80	815.90	–2.72	3.93
Sydney	–26.87**	11.90	–1,703.00**	796.60	–3.85	3.84
Westcoast	8.60	11.78	663.90	788.70	–0.96	3.80
Western Bulldogs	4.17	11.50	342.90	769.80	–0.12	3.71
Amateur League ^c^						
SANFL	–0.69	7.62	–26.62	510.20	–0.14	2.46
TAC Cup	1.19	6.66	105.00	445.90	2.07	2.15
WAFL	2.20	7.48	173.30	500.40	1.74	2.41
Constant	363.50***	85.60	21,123.00***	5,730.00	68.78**	27.59
Observations	907		907		907	
Adjusted R-squared	0.11		0.10		0.10	
F-Statistic	4.35		4.25		4.06	
Prob(F-Statistic)	0.00		0.00		0.00	
White test Prob > chi^2^	0.06		0.05		0.88	

^a^ the reference position is Defender

^b^ the reference team is Adelaide Crows

^c^ the reference league is Other.

*** p<0.01

** p<0.05

* p<0.1.

Drafting age is significant across all models, whereby for every additional year in the age at which they are first drafted, a player will play 3.22 fewer games, spend 212.60 fewer minutes on the field and receive 1.51 fewer BMV. This suggests that good players are generally recruited early on. Weight is positively associated with performance, with 3.80 games played for an additional kilogram, while height is negatively related to performance. Moreover, except for Sydney, all other drafting teams yield insignificant results. Relative to the Adelaide Crows, Sydney draftees play 26.87 fewer games over their careers.

The bidding system for F/S and C/A players yield insignificant results on all specifications. Conversely, the indigenous or non-indigenous player status is positive and highly statistically significant. This suggests that an Aboriginal and Torres Strait Islander player plays an additional 33.59 games relative to their equivalent non-indigenous counterparts. However, this variable is not statistically significant in the BMV model. This could indicate some form of discrimination where umpires fail to acknowledge the performance of Aboriginal and Torres Strait Islander players, consistent with Parsons et al. [38], who found biases in play-calling officials in Major League Baseball (MLB). However, the higher proportion of forwards among Aboriginal and Torres Strait Islander draftees compared to midfielders (who are more inclined to receive umpire accolades) may also contribute to this finding. Overall, the model confirms that a number of these factors (Indigenous or non-Indigenous, height, weight, midfield, and Sydney) are important determinants of player performance.

### Seasonal performance model

The seasonal performance model (Eq 2) is estimated using the panel dataset of 4,777 observations. The data comprise of listed player seasons of draftees selected from 2003 to 2016 and their respective performance from 2004 to 2017. The results are presented in Tables 6–8 for the three respective measures of performance. Given panel data, a fixed-effects model is well suited to account for the unobserved player characteristics. However, since most of the variables are time-invariant we chose to estimate a multilevel mixed-effects linear regression (mixed-effects model, columns 5, 8 and 11) that embeds both the fixed and random effects. As an intermediate step and to test the robustness of our results, we also estimate a random-effects model (columns 4, 7 and 10). One final concern with our modelling approach is that our estimates may be subject to sample selection bias because not all players are listed to play (the data for player performance was only provided for those who were listed in a team) in a season, with a few having retired and others delisted. Therefore, we estimate the mixed-effects model by adjusting for selection bias (columns 6, 9 and 12) using respective Inverse Mills Ratio (IMR) (see, e.g., Posso et al. [39]). In order to create this, we created a dummy indicator equal to 1 if a player played at least one game in the current season and 0 if they did not. Next, we estimate separate binary logit models with the dummy indicator as the dependent variable with the player’s age and one-period lagged performance as explanatory variables. The predicted outcomes from this model per player per season were used as the IMR. The respective IMR is then used as an additional explanatory variable in each of the three mixed effects model.

**Table 6:** Seasonal games played regression results.

Variables	Random Effects (4)		Mixed Effects (5)		Mixed Effects with Selection (6)	
	Games Played per Season					
	Coefficient	Std. Err	Coefficient	Std. Err	Coefficient	Std. Err
Drafting Age	–0.20	0.17	–0.18	0.19	0.11	1.16
Season Age	8.22***	0.42	8.23***	0.41	2.58***	1.50
Season Age^2^	–0.16***	0.01	–0.16***	0.01	–0.05***	1.01
Indigenous	1.41*	0.75	1.41*	0.84	1.11	1.69
Father–son						
≤ 2006	0.93	1.24	0.93	1.41	0.54	2.15
2007 ≤ 2014	1.60	1.24	1.65	1.39	1.19	2.16
≥ 2015	1.33	3.81	1.18	4.03	1.65	4.60
Club academy						
≤ 2014	0.30	2.02	0.23	2.22	–0.05	2.89
≥ 2015	1.78	1.88	1.60	2.01	2.04	2.77
Height	–0.14***	0.04	–0.13***	0.04	–0.11***	1.04
Weight	0.06**	0.03	0.05*	0.03	0.06**	1.03
Same Team as Drafting Team	1.45***	0.37	1.21***	0.36	0.83**	1.36
Traded	1.04*	0.58	0.980*	0.56	0.96*	1.56
Position Played ^a^						
Forward	–0.45	0.43	–0.45	0.48	–0.39	1.40
Midfielder	1.48***	0.44	1.45***	0.50	1.27***	1.41
Ruckman	–0.03	0.82	–0.03	0.92	–0.11	1.76
Drafting Team ^b^						
Brisbane	1.65*	0.95	1.73	1.07	1.06	1.89
Carlton	0.53	1.00	0.62	1.12	0.25	1.93
Collingwood	–0.31	1.01	–0.29	1.12	–0.37	1.93
Essendon	0.44	0.97	0.48	1.09	0.32	1.90
Fremantle	–0.49	0.99	–0.42	1.11	–0.51	1.92
Geelong	–1.28	0.98	–1.20	1.10	–0.97	1.91
Gold Coast	2.09*	1.21	2.16	1.34	1.48	2.12
Greater Western Sydney	1.30	1.15	1.33	1.28	0.79	2.07
Hawthorn	0.19	1.00	0.26	1.12	0.26	1.92
Melbourne	1.24	0.97	1.34	1.08	0.82	1.90
North Melbourne	–0.16	0.99	–0.14	1.11	–0.05	1.92
Port Adelaide	0.23	0.98	0.29	1.10	0.17	1.91
Richmond	0.23	0.98	0.27	1.10	0.01	1.91
St. Kilda	0.32	1.06	0.30	1.18	0.06	1.98
Sydney	–2.34**	1.03	–2.22*	1.15	–1.79*	1.96
Westcoast	0.36	1.01	0.35	1.13	0.21	1.94
Western Bulldogs	0.36	0.99	0.40	1.11	0.18	1.92
Amateur League ^c^						
SANFL	–0.28	0.66	–0.32	0.74	–0.23	1.61
TAC Cup	0.49	0.58	0.44	0.65	0.45	1.54
WAFL	–0.13	0.65	–0.17	0.73	–0.01	1.60
Inverse Mills Ratio					–34.52***	2.79
Constant	–69.48***	8.72	–70.72***	9.26	3.44	10.05
Observations	4,777		4,777		4,777	
Number of players	907		907		907	
R-Squared	0.20					
LR test			1,087.41		397.30	

^a^ the reference position is Defender

^b^ the reference team is Adelaide Crows

^c^ the reference league is Other.

*** p<0.01

** p<0.05

* p<0.1.

**Table 7:** Seasonal time on-field regression results.

Variables	Random Effects (7)		Mixed Effects (8)		Mixed Effects with Selection (9)	
	Time on Field per Season					
	Coefficient	Std. Err	Coefficient	Std. Err	Coefficient	Std. Err
Drafting Age	–19.35*	11.12	–17.38	12.50	2.12	10.61
Season Age	577.50***	28.62	580.30***	27.84	187.10***	33.75
Season Age^2^	–11.24***	0.61	–11.39***	0.59	–3.72***	0.70
Indigenous	104.10**	48.75	103.1*	55.95	83.08*	46.24
Father–son						
≤ 2006	52.57	80.67	51.41	93.07	25.17	76.48
2007 ≤ 2014	107.10	81.12	111.30	91.87	80.20	77.02
≥ 2015	100.70	252.50	90.25	268.90	120.80	240.80
Club academy						
≤ 2014	46.61	132.70	41.21	147.30	21.29	126.10
≥ 2015	118.70	124.50	105.90	134.10	133.80	118.60
Height	–7.68***	2.54	–6.96**	2.77	–5.15**	2.42
Weight	5.25***	1.92	4.546**	2.00	4.67**	1.83
Same Team as Drafting Team	100.00***	24.84	80.18***	24.44	53.23**	23.93
Traded	54.05	39.13	48.71	37.80	47.12	37.55
Position Played ^a^						
Forward	–48.02*	27.96	–47.80	31.77	–44.17*	26.54
Midfielder	67.22**	28.97	65.07**	32.91	52.16*	27.51
Ruckman	–83.64	53.55	–80.86	61.05	–89.03*	50.81
Drafting Team ^b^						
Brisbane	112.70*	62.10	117.90*	70.74	72.74	58.96
Carlton	30.07	65.30	35.64	74.20	11.82	61.98
Collingwood	–2.11	65.48	–1.76	74.56	–6.05	62.14
Essendon	40.87	63.33	43.62	72.20	33.55	60.09
Fremantle	–30.99	64.80	–26.41	73.71	–31.87	61.49
Geelong	–85.91	63.83	–79.91	72.84	–63.19	60.57
Gold Coast	152.20*	78.93	156.40*	89.21	110.70	74.97
Greater Western Sydney	95.41	74.80	95.84	84.98	60.17	71.02
Hawthorn	21.36	64.93	25.98	74.33	27.82	61.59
Melbourne	92.35	62.93	98.83	71.85	64.67	59.72
North Melbourne	–13.20	64.61	–11.66	73.54	–5.06	61.31
Port Adelaide	16.27	63.63	20.03	72.63	13.78	60.37
Richmond	19.17	63.79	21.08	72.70	4.50	60.53
St. Kilda	26.98	68.85	24.86	78.28	8.51	65.35
Sydney	–146.20**	67.24	–138.00*	76.44	–106.60*	63.84
Westcoast	33.46	65.74	32.59	75.05	23.20	62.37
Western Bulldogs	33.87	64.64	36.29	73.64	21.40	61.34
Amateur League ^c^						
SANFL	–13.40	43.12	–16.56	49.03	–10.17	40.92
TAC Cup	38.10	37.93	34.28	43.09	34.40	35.99
WAFL	–4.94	42.26	–7.98	48.08	3.13	40.11
Inverse Mills Ratio					–2,391.00***	120.40
Constant	–5,391.00***	577.00	–5,480.00***	617.70	–359.60	605.40
Observations	4,777		4,777		4,777	
Number of players	907		907		907	
R-Squared	0.21					
LR test			1,106.68		451.90	

^a^ the reference position is Defender

^b^ the reference team is Adelaide Crows

^c^ the reference league is Other.

*** p<0.01

** p<0.05

* p<0.1.

**Table 8:** Seasonal BMV regression results.

Variables	Random Effects (10)		Mixed Effects (11)		Mixed Effects with Selection (12)	
	Brownlow Votes per Season					
	Coefficient	Std. Err	Coefficient	Std. Err	Coefficient	Std. Err
Drafting Age	–0.37***	0.06	–0.35***	0.07	–0.32***	0.07
Season Age	1.88***	0.19	1.95***	0.18	1.33***	0.22
Season Age^2^	–0.03***	0.00	–0.04***	0.00	–0.02***	0.00
Indigenous	0.24	0.26	0.26	0.32	0.22	0.32
Father–son						
≤ 2006	0.27	0.43	0.23	0.53	0.18	0.52
2007 ≤ 2014	0.55	0.45	0.55	0.53	0.51	0.53
≥ 2015	–0.82	1.53	–0.74	1.64	–0.71	1.62
Club academy						
≤ 2014	0.07	0.76	0.02	0.87	–0.02	0.86
≥ 2015	–0.08	0.74	–0.10	0.81	–0.08	0.80
Height	–0.04**	0.01	–0.03*	0.02	–0.03	0.02
Weight	0.05***	0.01	0.05***	0.01	0.05***	0.01
Same Team as Drafting Team	0.82***	0.16	0.71***	0.16	0.64***	0.16
Traded	–0.30	0.26	–0.33	0.25	–0.34	0.25
Position Played ^a^						
Forward	0.34**	0.15	0.31*	0.18	0.31*	0.18
Midfielder	1.79***	0.16	1.68***	0.19	1.66***	0.19
Ruckman	–0.10	0.29	–0.12	0.35	–0.13	0.35
Drafting Team ^b^						
Brisbane	–0.70**	0.34	–0.65	0.41	–0.71*	0.40
Carlton	–0.02	0.36	–0.02	0.43	–0.05	0.42
Collingwood	–0.42	0.36	–0.41	0.43	–0.42	0.42
Essendon	–0.52	0.34	–0.44	0.42	–0.46	0.41
Fremantle	–0.23	0.35	–0.19	0.43	–0.20	0.42
Geelong	–0.63*	0.35	–0.58	0.42	–0.55	0.41
Gold Coast	–0.35	0.44	–0.33	0.52	–0.40	0.51
Greater Western Sydney	–0.13	0.41	–0.10	0.49	–0.16	0.49
Hawthorn	–0.29	0.35	–0.29	0.43	–0.28	0.42
Melbourne	–0.60*	0.34	–0.52	0.41	–0.57	0.41
North Melbourne	–0.45	0.35	–0.41	0.43	–0.40	0.42
Port Adelaide	–0.50	0.34	–0.45	0.42	–0.45	0.41
Richmond	–0.08	0.35	–0.08	0.42	–0.11	0.41
St. Kilda	–0.41	0.38	–0.37	0.45	–0.40	0.45
Sydney	–0.29	0.37	–0.25	0.44	–0.19	0.44
Westcoast	–0.40	0.36	–0.33	0.43	–0.35	0.43
Western Bulldogs	–0.15	0.35	–0.09	0.43	–0.11	0.42
Amateur League ^c^						
SANFL	0.00	0.24	–0.01	0.28	–0.01	0.28
TAC Cup	0.390*	0.21	0.35	0.25	0.35	0.25
WAFL	0.28	0.23	0.24	0.28	0.25	0.27
Inverse Mills Ratio					–3.80***	0.80
Constant	–15.46***	3.43	–17.33***	3.74	–9.246**	4.07
Observations	4,777		4,777		4,777	
Number of players	907		907		907	
R-Squared	0.18					
LR test			1,267.19		1,173.47	

^a^ the reference position is Defender

^b^ the reference team is Adelaide Crows

^c^ the reference league is Other.

*** p<0.01

** p<0.05

* p<0.1.

Like the career model, the results for both games played and time on field per season mimic each other. Both season age and the quadratic component (a proxy for player’s experience) are statistically significant. The effects of all other variables remain consistent with those in the career model, apart from drafting age which is statistically insignificant in both games played and time on field models. However, it remains statistically significant in the BMV model (column 12). For example, with each additional year at which the player was drafted, seasonal BMV drops by 0.32. Being traded to another team prior to the start of the season (traded), in general, does not affect performance except for the number of games played (at the 10% level of significance). The statistical significance of the IMR in all three tables provides evidence of selection bias and justifies using the sample selection model. Again, the model based on season performance confirms that many of the included factors are important determinants of player performance.

### Predicted performance

**Fig 2:**
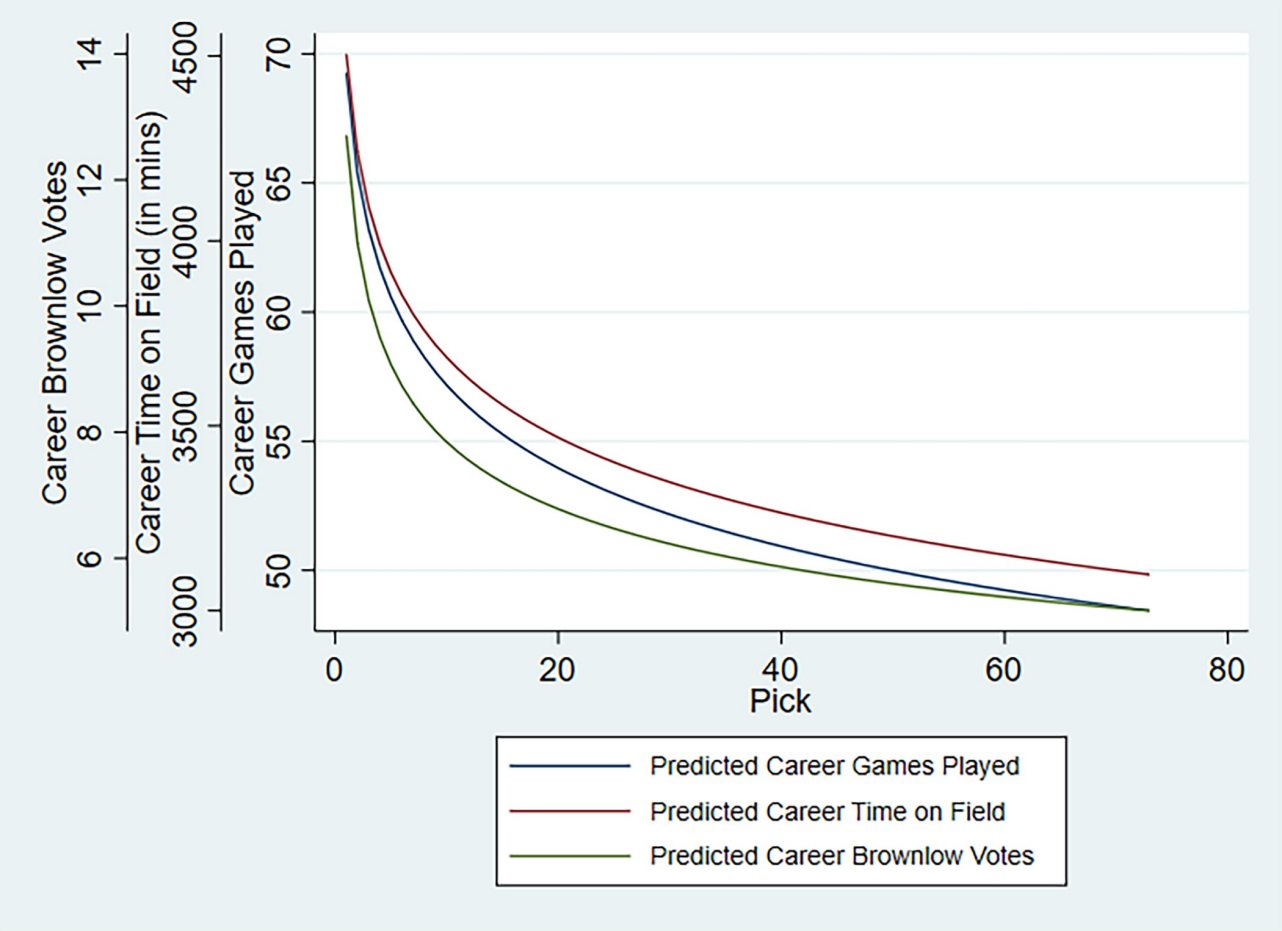
Predicted average career performance per pick.

**Fig 3:**
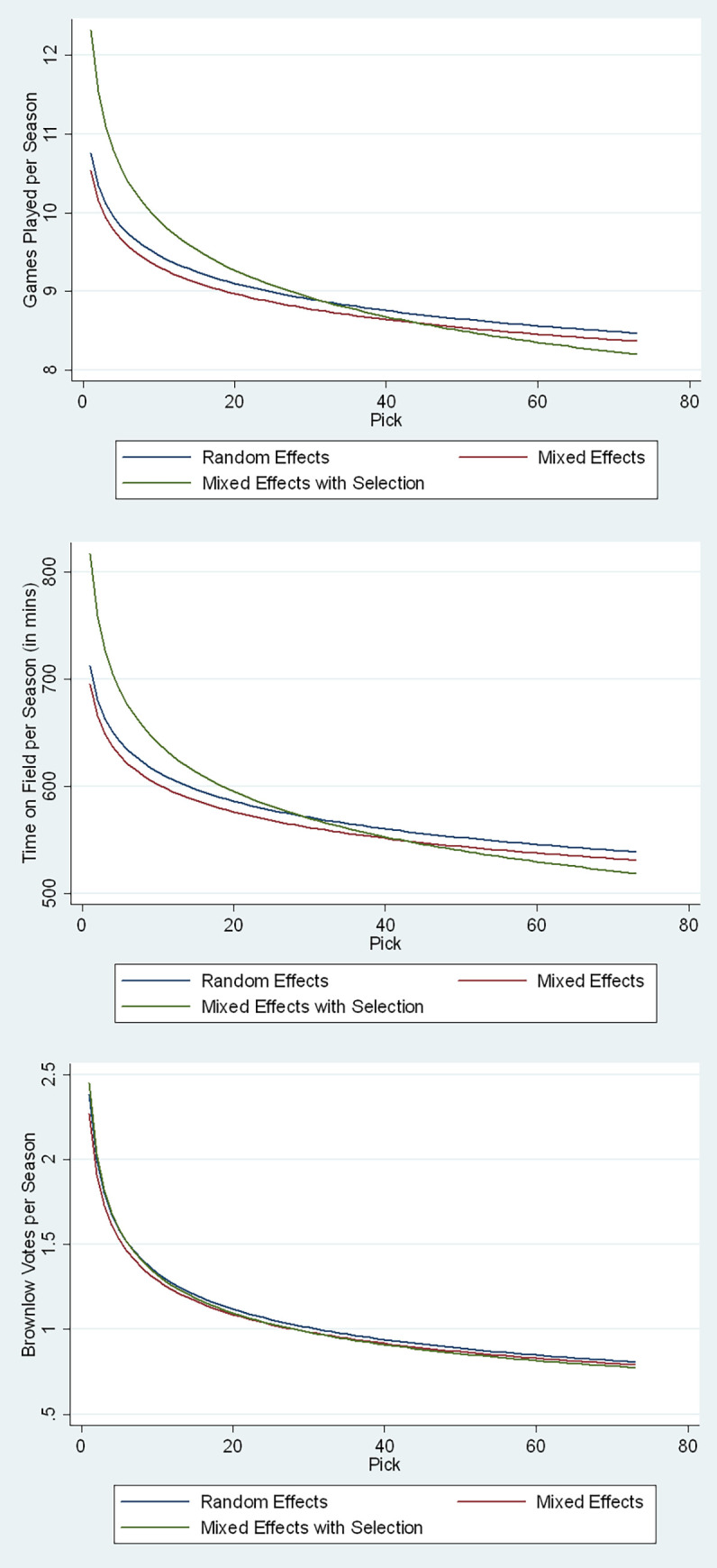
Predicted average seasonal performance per pick.

Using the respective estimated models, we next predict players’ performance for each of the three different measures (games played, time on field and Brownlow Votes) using the regression results obtained from Tables 5–8. We then average the predicted performance for both career and season by pick, which allows us to determine the players’ performance-pick relationship. Upon observing the trend, we use a power model (similar to a conventional production function) to fit the trend of the performance-pick relationship as follows:

$$Average\;Predicted\;Performance\;per\;Pick=\beta_{1}{Pick}^{\beta_{2}}$$ (3)

where β_1_ and β_2_ are unknown parameters. Transforming Eq 3 into logarithmic form (e.g., ln *(Average Predicted Performance per Pick)* = ln *β*_*1*_ + *β*_*2*_ ln *Pick*), enables us to estimate it using OLS (the results can be obtained from the authors upon request.). After individually estimating Eq 3 for all three performance measures we plotted the trend lines for both the predicted career (Fig 2) and seasonal (Fig 3) estimations.

As expected, we find a downward sloping monotonic relationship between predicted performance and pick. Fitted career games and time on field fall at a relatively lower rate with respect to pick than does career BMV (see Fig 2), like the trend observed in the raw data in Fig 1. This could be because the number of BMV distributed per game is capped, and as explained earlier it reflects the quality of the game played by a player as judged by an umpire. Hence, when comparing the two, we would conclude that even though the disparity in game time between players recruited at varying pick numbers could be minimal (roughly 30%), the quality of their output could vary significantly based on their selection as represented by the BMV.

Similar trends are observed with performance per season (see Fig 3). As the seasonal panel data is restricted to listed player seasons both the random-effects and mixed-effects models yield much flatter curves compared to career performance. However, once a correction for selection bias is performed, the slopes (green line in Fig 3) mimic those of the career performance models.

## The new DVI

The current AFL DVI, as mentioned earlier, was created by regressing career player salaries of draftees selected over 15 years. We argue that draftee payments do not necessarily reflect their performance. The main purpose of this paper is to create a DVI based on post-draft performance and compare it with the current AFL DVI.

**Fig 4:**
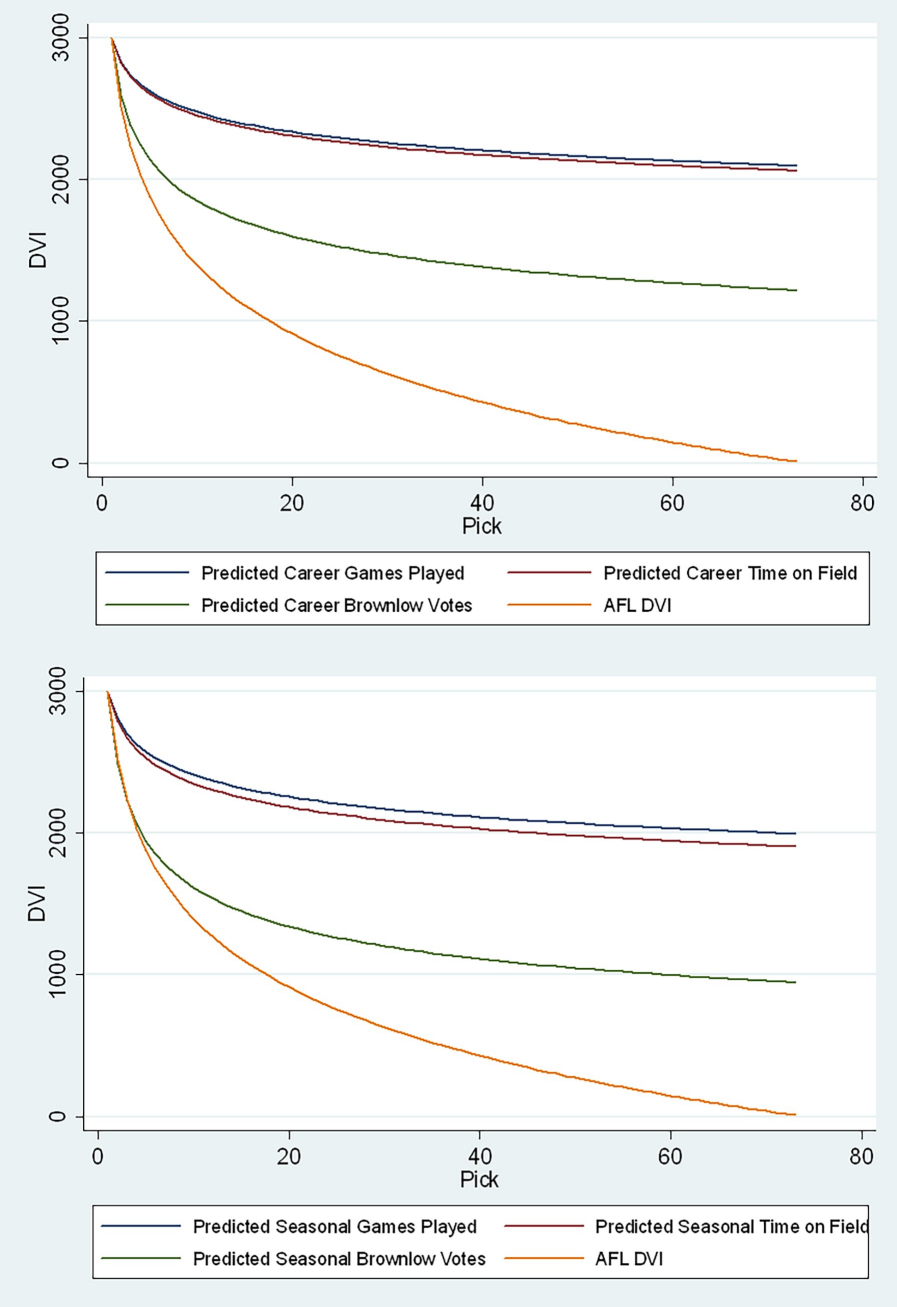
Current and predicted DVIs based on career & seasonal predictions.

To recap, we have modelled performance using three indicators in both a player’s career and individual seasons. The predicted outcomes are then averaged per pick and fitted using a power model. However, as all three performance measures used in this paper have different scales, comparing them to the AFL DVI proves difficult. Hence, the predicted career and season (here we used predictions from the mixed-effects model adjusting for selection bias.) performance are retrospectively scaled to the AFL’s DVI as presented in Fig 4. As the AFL DVI ranges from 3,000 points at pick 1 to 9 at selection 73, the fitted outcomes are scaled to the same values.

By plotting the above-mentioned equations where *0 < Pick <74*, we can observe two varying phenomena (Fig 4). The expected games per season and time on field DVIs decline at a much lower rate when compared to the AFL’s DVI, while the BMV-based DVI falls more steeply, somewhere between the two.

As an example, the total variation between the first and seventy-third pick in the expected games and time models is about 30%, while the BMV-based DVI and the current DVI sit at 87% and 99%, respectively. At first glance, the slower decline and much flatter value function observed in the games played and time on field performance metrics bring into question the veracity of the proposed models. However, as advised earlier, game time indicators not only represent the performance of a player but also include the competing motives of decision-makers who intend to justify their decisions (commonly referred to as sunk investment plays). Previous literature suggest that games played is not an accurate indicator of draftee performance [37]. Other authors have instead used player ranking points [23] and contribution to margin [31]. In contrast, the BMV model provides a better indicator of performance because it does not simply look at a player’s game time but his contribution instead.

Our findings clearly show a mismatch between performance and player salary in the AFL. Similar to the PVC in the NFL, the surplus (difference between performance and payments) increases with the progression of the draft [40]. The purpose of the draft is to assist teams that perform poorly to procure the best amateur talent and thereby increase their winning prospects. Given the declining performance of players recruited as the draft progresses, early picks still outperform their counterparts. However, since the difference between the AFL’s DVI and expected performance grows through the draft, in a purely financial sense, it is in the club’s best interest to trade down to increase their surplus [31].

The AFL DVI was primarily created to value the selections that teams had to forgo when nominating F/S and C/A players. While this new bidding system proved to be fairer than the previous rules, the number of draftees recruited under these systems has decreased since 2015. Since teams tend to weigh the picks that they forgo when they enforce the PAS rules, especially if such a player is contested in the first round, some forgo matching a bid for a player due to the high price of the selection. While Chandrakumaran [41] concluded that there was no observable incentive for a team to intentionally lose end of season games in the post-DVI era of the AFL, the need for clubs to accumulate DVI points to accommodate foreseeable PAS could potentially create a perverse incentive. Also, a simple analysis of pick-to-pick trades in the league suggests that clubs use a different scale to value selections, as the profit on trades using the DVI shows no obvious trend because the motivation behind a trade would dictate the value exchanged. Hence, utilising the proposed models in lieu of the AFL’s DVI should prove to be more equitable, as teams would use projected performance as the consideration in a trade [31].

## Conclusion

The primary purpose for the AFL to create a DVI was to introduce a pricing structure so that the league could recuperate value from teams who utilise the F/S and C/A rules. However, the choice of player payments as the determinant of value has been found to be inadequate when compared to the alternative models presented in the literature [23, 32, 36] while also possibly creating arbitrage opportunities [31] and perverse incentives [41].

In this paper, we estimated various models for player performance using alternative measures of performance, namely, number of games played, time on field and Brownlow Medal Votes. We then used predicted players’ performance to construct respective DVIs. We found that games played and time on field based DVIs both declined at a much lower slower rate. These findings are consistent with competing interests that selectors have in ensuring their recruits perform (or at least appear to have performed) to validate their sunk investments. The flatness of the games played-based DVI questions the effect the draft has on the league in disseminating amateur talent to teams since there is not much variation in the time players spend on field relative to the pick used to recruit them. A similar question is raised by Motomura et al. [42] in the NBA and they conclude that a team’s road to success is defined more by the effectiveness of their management and not necessarily by the number of good selections in the amateur draft.

In comparison, the BMV-based DVI proved to be more adequate because it characterises a player’s actual contribution as adjudged by an umpire. Yet, this index has a flatter slope than the AFL’s current DVI, suggesting that the latter may not adequately reflect actual performance, and its use within the context of other competitive balance policies will not necessarily be effective. Instead, a BMV-based DVI is highly recommended as it defines draftee value in terms of actual output instead of what a team is willing to pay to secure that player (which can be based on a variety of factors). Utilising the example of Jack Steele, had the league used our proposed alternative (Table 8, season estimates, BMV), Greater Western Sydney would still use pick 14 (1,476 points) by sacrificing the 23^rd^ (1,291 points) selection which they had at the time. However, unlike under the AFL’s DVI rule, where their 24^th^ pick was substituted by pick 27, to cater for the difference in values between selections 14 and 23, they would be given the 43^rd^ pick instead. Furthermore, given that the proposed DVI declines at a much slower rate than the current AFL’s DVI, the 20% discount given on bidding ascensions for picks in the first round need not be entertained as well (had the discount been added, pick 24 of Greater Western Sydney would be replaced by 18).
